# Supplementary material for: Second-dose measles vaccination coverage and associated factors among children aged 19 to 35 months in Debre Birhan city, Ethiopia, 2024: A community-based cross-sectional study
Source: PLoS One. 2026 Feb 26;21(2):e0342501. doi: 10.1371/journal.pone.0342501 (PMC12944772; doi:10.1371/journal.pone.0342501)
Supplement: Attachment — Submitted filename: Response to Reviewers.docx [file pone.0342501.s002.docx]

**Response to Reviewers**

1. **Journal Requirements:**
   1.1 Please ensure that your manuscript meets PLOS ONE's style requirements, including those for file naming.

**Response:** Thank you, after reviewing the PLOS ONE style templates, we revise the whole document, e.g., we use Level 1 heading for all major sections (Abstract, Introduction, Materials and methods, Results, Discussion, etc.) with bold type, 18pt font. We also Cite figures as “Fig 1”, “Fig 2”. We use bold type for the figure titles.

1.2 In the ethics statement in the Methods, you have specified that verbal consent was obtained. Please provide additional details regarding how this consent was documented and witnessed, and state whether this was approved by the IRB

**Response:** Thank you, we provide an additional detail regarding how the verbal consent was documented and witnessed (see the Ethics approval and consent to participate on page 6 and 7)

1.3 We note that your Data Availability Statement is currently as follows: “All relevant data are within the manuscript and its Supporting Information files.” Please confirm at this time whether or not your submission contains all raw data required to replicate the results of your study. Authors must share the “minimal data set” for their submission. Authors do not need to submit their entire data set if only a portion of the data was used in the reported study.

**Response:** Thank you, we share the data set.

1.4 Please amend either the abstract on the online submission form (via Edit Submission) or the abstract in the manuscript so that they are identical [Note: HTML markup is below. Please do not edit.]

**Response:** Ok thanks we edit during submission

2. **Response to reviewers' comments:**
   2.1 **Response to Reviewer #1 comments**

**2.1.1** The discussion section should present the evidence obtained, as well as exploring and showing possible strategies to increase coverage of two-dose measles vaccination (19 and 35 months). These strategies should be based on data from other literature sources, as well as the findings from this study.

**Response:** We sincerely appreciate your insightful and helpful comments. We revise the discussion part as your recommendation, see line 324 to 332

2.1.2 The Conclusion section presents recommendations based solely on the data from this study. The study would have been more comprehensive if the factors affecting vaccination coverage had been categorized, clearly listed, systematized and described in terms of practical guidance for the health worker. For instance, Group 1 encompasses priority factors that were identified on the basis of the study conducted, with consideration given to reliable results on coverage with two doses of measles vaccination. Group 2 - secondary (unreliable results from work) factors that require the development of strategies to adjust and modify them in order to positively influence vaccination coverage. In particular, this could be the promotion of conclusions about the positive effects of vaccination during pregnancy planning consultations, antenatal care and postnatal care; communication with patients seeking counselling for any medical intervention related to the child, and so on.
      This will improve the quality of the study and expand potential interventions to increase vaccination coverage in real-world practice, including in the context of later timing of vaccination against other infectious diseases.

**Response:** Thanks for your comments, we revise it as recommended, see the conclusion section from line 339 to 345.

**2. 2 Response to reviewer #2 comments**

1. The authors present an interesting topic but there are a couple of issues in the submitted manuscript. These relate mainly to sampling design, analysis and discussion sections. These should be addressed before it can be recommended for publication.

**Response:** Thank you very much for reviewing our paper and for your insightful comments, and recommendations. We see it critically and revise accordingly.

2. **BACKGROUND:**
   a. **L61** - the mortality numbers quoted are modelled estimates. They are NOT reported deaths. And authors should quote the latest estimates published Nov 2024.

**Response:** Thank you very much, we use the latest estimate published on 14, Nov, 2024, see line 59 to 63.

b. **L64** - Number of deaths prevented by MCV between 2000 and 2021 is based on a comparator of NO Vaccination not at existing rates of vaccination between 2000 and 2021.

**Response:** We correct it as your recommendation, see line 64 and 65.

c. **L75-76** -- The sentence reads as if ETH Ethiopia introduced the first dose of the measles vaccine in 2019. In fact, ETH introduced (MCV1) in 1980 and the second dose (MCV2) in February 2019.

**Response:** Thank you for your comment, and we rewrite it as “In early 2019, the Expanded Program on Immunization (EPI) began administering the measles two vaccine at 15 months to ensure adequate protection**.”**

3. **METHODS:**
   a. **L105 7 ff** -- Population universe - It is unusual to have a population-based listing of all 19–35-month-old children by household. How was this last made available to the authors? Was it from a special service area of a model health center or medical college? In that case, this population may not be representative of general population of ETH for this age group and the conclusions may not be generalizable. How was the base population list by household generated in the first place? Was it done through periodic community census or was it done through attendance at immunization clinics by the pregnant mother or the infant? If done by the latter method, this could introduce a serious selection bias to the results as the infants/children who did not attend immunization clinics could have been missed from the base population list and result in higher than true proportion of children immunized.

**Response:** Thank you for your insightful comments. As described in our background, “In late 2023 and early 2024, Debre Birhan City, Ethiopia, experienced two measles outbreaks, despite a reported MCV1 vaccination coverage of 90–95% among children aged 19–35 months.” In response to these outbreaks, the Debre Berhan City Health Bureau conducted a census in each Kebele through health extension workers. The gross data, specifically the number of children aged 19–35 months, were obtained from this census.

b. **L105-106:** The authors studied 19–35-month-old children in May 2024. Then the authors were studying the birth cohort born between May 2021 to Dec 2022. Authors should clearly state this and mention the date cut-offs if applied.

**Response:** We accept the comment and we state it, see line 103-104

c. **L127**: Definition of dependent variable MCV2-By definition all MCV2 positive children must have received MCV1. The authors should clearly state that they ascertained MCV1 status too when they categorized a child as MCV2 or if they followed some other methods to determine MCV2 status.

**Response:** Thank you for your comment**,** even if we didn’t state it under dependent variable as you stated above, we include MCV1 as a prerequisite for MCV2 under the operational definition of MCV2- see line 139 and 140

d. **L133**: Of all the independent or explanatory variables, MCV1 is unique as it is on direct pathway to MCV2+ status, and hence could be a confounder. Hence MCV1 cannot be considered an explanatory variable as an MCV1-negative status would preclude MCV2+ status. Also, L140: Authors do not state clearly that they verified that every MCV2 positive child had indeed received the second dose of MCV and not just a delayed first dose of MCV. Please see more on this in RESULTS.

**Response:** Thank you very much for your insightful comments. We apologize for the technical oversight. We included MCV1 as an independent variable based on the conceptual framework principle—where variables are aligned with questionnaire items. However, in practice, MCV1 was not analyzed as an independent variable in the bivariable logistic regression. To prevent any potential misinterpretation, we have now removed MCV1 from the list of independent variables.

e. **L146 and L154-55**: Perception and awareness scores: What was the value of scores that were used to determine "good perception" and "high awareness"?

**Response:** Thank you, as we describe on operational definition “participants who scored at or above the mean were classified as having a good perception and participants who scored above or equal to the median on the seven awareness questions were considered to have a high level of awareness.

4. **RESULTS:**
   a. **Table-1** - Income currency to be mentioned.

**Response:** Thank you, corrected-see table 1

b. **Table 2: MCV1 status** - 13% of MCV2 children are stated to be MCV1 negative. By definition they cannot be MCV2+, at most they would be delayed MCV1. These children should have been excluded from all further analysis or replacement samples collected.

**Response:** Thank you for your insightful observation. We fully agree that, under routine immunization guidelines, a child should not receive MCV2 without first receiving MCV1. We also acknowledge that, based on this standard, these children could have been excluded from the analysis.

However, in our study, we intentionally included them for the following reasons:

- They were **eligible for MCV2 by age**, regardless of their reported MCV1 status.
- We assumed that **excluding them might underestimate the real-world coverage gap** of MCV2 and would not reflect programmatic realities.
- **Including them provides a more accurate picture of operational challenges**, such as documentation gaps, caregiver recall issues, and deviations in service delivery, which are common in low-resource settings.
- Furthermore, during our proposal development and literature review, we found that other studies had taken a similar approach by including children reported as MCV1-negative in MCV2 coverage analyses. For example:

1. [Second dose measles vaccination coverage and associated factors among children aged 24–35 months in Sub-Saharan Africa](https://www.frontiersin.org/journals/publichealth/articles/10.3389/fpubh.2022.1029740/full) – *Frontiers in Public Health, 2022*
2. [Assessment of second dose measles vaccination coverage using the 2016 Ethiopia Demographic and Health Survey](https://www.afenet-journal.net/content/article/6/1/full/) – *Pan African Medical Journal, 2023*
3. <https://pdfs.semanticscholar.org/713b/74af62b0e5206efd6ac8eede268d4cd6408d.pdf>

Nonetheless, we sincerely appreciate your thoughtful comment and insights. If re-analysis is strongly recommended, we are willing to proceed accordingly

c. **L238-239:** Children vaccinated for any vaccine (YES/NO) - By definition no MCV1 negative child can be MCV2 positive and therefore including them in the analysis biases all estimates. The entire analysis should be redone after excluding these MCV1 negative children.

**Response:** Thank you very much for your insightful comment. We agree with your observation that, by definition, a child who did not receive MCV1 cannot receive MCV2. In our dataset, all children who were marked as MCV1-negative were also MCV2-negative. Therefore, no MCV1-negative and MCV2-positive cases were included in the analysis. We have now clarified this point in the manuscript under table -2 by stating “All children recorded as MCV1-negative were also MCV2-negative; thus, no inconsistencies in the MCV1–MCV2 sequence were present in the dataset” to avoid any misinterpretation.

d. **T5:** Rows comparing Child received any vaccination vs. MCV2 status actually shows that a child not receiving any vaccine had higher odds (28/11) of getting MCV2 than those with any vaccine (239/348). The AOR would therefore be the inverse of 3.04. However, as stated above including MCV0 children in prior vaccination status in the sample is erroneous as they can never become MCV2. The analysis should be redone.

**Response:** Thank you for your valuable comment. We fully agree with your observation. The issue you noted was due to a typographical error that occurred when transferring the multivariable table output. Specifically, the categories for MCV2 vaccination status were inadvertently reversed: MCV2 vaccinated was labeled as not vaccinated, and vice versa. To clarify, based on the correct cross-tabulation from our dataset, children who did not receive any vaccine had a lower odds of receiving MCV2 (11/28 = 0.393) compared to those who had received at least one vaccine (348/239 = 1.456). The correct cross tab output table is provided below for reference and we correct in revised manuscript (see Table -5).

| **Does your child had vaccinated for any vaccine? * 604. MCV2 Crosstabulation** | | | | |
| --- | --- | --- | --- | --- |
| Count | | | | |
|  | | 604. MCV2 | | Total |
|  |  | No | Yes |  |
| 303.Does your child had vaccinated for any vaccine? | Yes | 239 | 348 | 587 |
|  | No | 28 | 11 | 39 |
| Total | | 267 | 359 | 626 |

**4: DISCUSSION**:

5.1 L265: Please mention country of GONDAR. Please rewrite the discussion section in light of revised analysis.

**Response:** Thank you for your comments we correct accordingly (see Discussion section Line 270, 284,301).
