# Supplementary material for: Second-dose measles vaccination coverage and associated factors among children aged 19 to 35 months in Debre Birhan city, Ethiopia, 2024: A community-based cross-sectional study
Source: PLoS One. 2026 Feb 26;21(2):e0342501. doi: 10.1371/journal.pone.0342501 (PMC12944772; doi:10.1371/journal.pone.0342501)
Supplement: Attachment — Submitted filename: Comment for PLO, Me vaccine.docx [file pone.0342501.s004.docx]

**Title:** Why you select only second dose vaccine coverage:

**Abstract:**

- **Line 33:** “May 10 to 30, 2024” → “May 10–30, 2024” (use en dash).
- Ensuring consistency in describing the participants as children (not caregivers) and using precise language for the age range and sampling scope, I have assumed "in all over the paper" means applying this uniformly throughout the manuscript for clarity as a systematic random sampling method was used to recruit 626 children aged 19-35 months.
- **Line 43:** “Significant factors associated with the second dose of measles vaccination were;” → “Significant factors associated with second-dose measles vaccination coverage were:”
- Significant factors associated with receipt of the second dose of measles vaccination included father's education level (primary, secondary, or college). However, as expected, these sociodemographic variables are known to promote higher vaccination coverage in educated families. This is a well-documented and almost universally true finding in global health literature. In other words, higher parental education is generally associated with greater health literacy, better access to health information, and increased ability to navigate healthcare systems." It is not a novel discovery. Reporting it without deeper context or specific relevance to the local setting can make the study seem unoriginal and its conclusions overly simplistic.

**Possible recommendation to improve it**: To strengthen the impact of your study, contextualize the finding locally by explaining why father’s education specifically matters in Debre Birhan-such as the high proportion of male-headed households and cultural decision-making dynamics-and highlight what is new in your setting: emphasize that maternal education was not a significant predictor, unlike in many other studies, which underscores the unique and pivotal role of fathers in this community. Use stronger analytical language by framing the education effect as a clear “dose-response relationship,” where higher paternal education corresponds to progressively higher odds of vaccination.

Then, make actionable, father-focused recommendations, moving beyond generic awareness-raising to propose targeted strategies such as workplace outreach, male-tailored health messaging, and involving fathers directly in immunization visits. Finally, reframe the discussion to present father’s education not merely as a well-known sociodemographic factor, but as a key lever for designing locally effective, gender-sensitive interventions in Debre Birhan that can meaningfully improve second-dose measles vaccine coverage

- **Line 49:** “Therefore, to improve coverage, it is essential to strengthen health education, streamline vaccination schedules, and enhancing communication with parents are essential to boost coverage.” → This is repetitive. Simplify to: “Therefore, to improve coverage, it is essential to strengthen health education, streamline vaccination schedules, and enhance communication with parents.”
- To fully address measles prevention, I am strongly recommended to assess vaccine effectiveness in addition to coverage. This will help determine whether waning immunity or primary vaccine failure, key challenges in current measles control, are contributing to ongoing outbreaks.
- Other significant predictors included whether the child had received any prior vaccine, whether any vaccination schedules had been canceled or postponed, as well as caregivers’ levels of awareness and perception regarding measles vaccination. All of these are established factors known to influence vaccination uptake
- Why you include those who canceled or postponed vaccination schedules as study participants.
- How the one who took another vaccine before increases the vaccination coverage of measle vaccine?

**Introduction:**

- Well-written and clear. Good context and rationale.
- **Line 90–93:** Sentence: “This also raise concerns…” → “This raises concerns…”
- Ensure consistency in referencing: e.g., “[6, 8]” in revised manuscript (line 75) is fine.

**Methods:**

- **Study Setting:** Well described.
- **Sample Size Formula (Line 115):** “P = 0.48%” should be “P = 0.48” (without % symbol). Also, add a space after “0.48”.
- **Sampling:** Clear and reproducible.
- **Line 145:** “In this study, parental recall was used for 12 out of 626 children (1.9%).” → Good addition.
- **Operational Definitions:** Clear, but ensure **Awareness** and **Perception** scoring is explicitly referenced (mean/median).
- **Ethics:** Well documented.

**Results:**

- **Tables:** Ensure all table titles are consistent (e.g., Table 1, 2, 3, 5).
- **Figure 2 Title:** “Measles two average” → “Measles second-dose coverage”
- **Line 225–227:** The coverage is stated clearly. Figure 2 is simple but acceptable.
- **Table 5:** Ensure column alignment. “Not vaccinate” → “Not vaccinated”.
- **AOR Reporting:** In text (Lines 248–258), ensure confidence intervals are formatted consistently: “(95% CI: 1.17–3.69)”.

**Discussion:**

- **Line 283–284:** “Gondar, Ethiopia (75.68%)” → Consider citing the correct reference.
- **Line 285–286:** “The disparities noted, especially in China and JapanThe disparities noted, especially in China and Japan,” → Duplicate text. Remove repetition.
- **Line 301–305:** Well articulated.
- **Line 332–333:** “Ethiopia; Gondar,” → “Ethiopia, including Gondar,”
- **Line 339–347:** The paragraph on strategies is relevant but could be more concise. Consider integrating into conclusion.

**Limitations:**

- Clearly stated. You may add: “Recall bias was minimal given only 1.9% relied on recall.”

**Conclusion and Recommendations:**

- Clear and practical.
- **Line 358–365:** Repetitive with abstract. Ensure concise, actionable recommendations.
- Suggest specifying: “Targeted health education for fathers, reminder systems for appointments, and community engagement.”

**References:**

- Formatting is inconsistent (some with full URLs, some without). Follow PLOS ONE citation style.
- Ensure all hyperlinks are properly bracketed and accessible.
- Example: Reference 1“Measles cases surge worldwide…” URL is very long; consider shortening or using DOI if available.
- Check for duplicate or misplaced references (e.g., references 6 and 8 are same?).

**General Comments:**

- **Language:** Mostly clear but some long sentences. Break them for readability.
- **Typos:** “overage” → “coverage” in Figure 2.
- **Consistency:** Use “MCV2” throughout instead of “measles two”.
- **Figures/Tables:** Ensure all are cited in text and legends are clear.
- **Response to Reviewers:** Well addressed. Ensure all changes are reflected in the main manuscript (some tracked changes remain).

**Recommended Actions Before Submission:**

1. Run a spell-check and grammar check.
2. Standardize reference formatting.
3. Verify all numbers in tables match text.
4. Consider removing Figure 2 if space is limited
