# Supplementary material for: Second-dose measles vaccination coverage and associated factors among children aged 19 to 35 months in Debre Birhan city, Ethiopia, 2024: A community-based cross-sectional study
Source: PLoS One. 2026 Feb 26;21(2):e0342501. doi: 10.1371/journal.pone.0342501 (PMC12944772; doi:10.1371/journal.pone.0342501)
Supplement: Attachment — Submitted filename: Response_to_Reviewers_auresp_3.docx [file pone.0342501.s005.docx]

**Response to Reviewers**

Manuscript

PONE-D-24-57768R1: Second-dose measles vaccination coverage and associated factors among children aged 19 to 35 months in Debre Birhan city, Ethiopia, 2024: a community-based cross-sectional study

Reviewer #3:

1. Abstract: Please specify that the adjusted odds ratios were those associated with having received the second dose of measles vaccine. Significant factors associated with the second measles vaccine dose were …. Need a little more work to clarify language in the results section of the abstract

**Response:** Thank you for your insightful comment and suggestions, we correct it as Significant factors associated with the second dose of measles vaccination were…….

2. Introduction: this describes the issue clearly and presents an excellent rationale for this research.

**Response:** Thank you for acknowledging our rationale, which clearly reflects the motivation that led us to conduct this research.

3. **Methods**: Excellent description of study setting and the study sampling methodology is very sound and clearly described.

**Response**: We sincerely appreciate your positive evaluation of our description of the study setting and the methodological rigor of our sampling approach.

**Specific comments:**

1. If vaccination was not recorded on the infant immunization card or the health card, the parent was asked to recall whether measles two vaccination had been given or not (Line 144)
   1.1 Can such parental recall be justified? For what proportion was this the method used to determine receipt of vaccine.

**Response:** Thank you for raising this important point. In our study, vaccination status was primarily verified using the infant immunization card or health card. However, in cases where these records were not available, parental recall was used as an alternative source of information. Parental recall has been commonly employed in similar population-based surveys, including the Demographic and Health Surveys (DHS), and has been shown to provide reasonably reliable estimates in the absence of written records. Even though this we acknowledge it in limitation section. In our study, parental recall accounted for -12/626=1.917 % of responses, we have now clarified this in the Methods section, Line 145. “In this study, parental recall was used for 12 out of 626 children (1.9%).

1.2 Was there a way that vaccination status was verified with the concerned health authorities?

**Response:** Thank you for this insightful comment. In our study, vaccination status was assessed using immunization/health cards and, when unavailable, caregiver recall. Direct verification with health facility or health authority records was not conducted, as our study was community-based and designed to capture information at the household level, similar to the methodology used in national Demographic and Health Surveys (DHS) and WHO immunization coverage surveys. We accept it, we put as limitation of this study and we state under limitation of the study as facility-level verification could have further strengthened the accuracy of vaccination status, see line 334-339.

**Results**

1. Good clear description of socio-demographic characteristics of mother-child pairs. Table, change ‘Categoties’ to ‘Categories’

**Response:** Thank you for your positive feedback on the description of the socio-demographic characteristics of mother–child pairs. We have corrected the typo in the table header, changing “Categoties” to “Categories.”

2. Table 2: Move the variables reporting parity and birth order and where child living to Table 1

**Response:** Thanks for your comment we move it to Table 1

3. Figure 2 legend: change ‘Figure 2: Measles two overage among children’ to’ Figure 2: Measles two coverage among children’

**Response:** thanks, we correct the error

4. Table 3 change ‘Ever has been post-pond or cancelled for vaccination’ to ‘Ever had a vaccination postponed or cancelled’

**Response:** thank you for your comment and we change it to “Ever had a vaccination postponed or cancelled”.

5. Change ‘Among these, five factors remained significant in the multivariable logistic regression analysis’ to ‘Among these, five factors remained independently associated with having received MCV2 in the multivariable logistic regression analysis’

**Response:** Thank you for your comment we correct it as ‘Among these, five factors remained independently associated with having received MCV2 in the multivariable logistic regression analysis….’

6. Change ‘Those children whose father’s educational status was primary, secondary, and college & above increased their odds of vaccinating’ to ‘In comparison to children whose fathers who had not received formal education, children whose father’s educational status was primary, secondary, and college & above had an increased odds of receiving MCV2’

**Response:** Thanks, we correct it accordingly, see line 248 to 250

7. Change ‘Mothers who had no schedules cancelled/postpone increased their odds of vaccinating their children for MCV2 by five times compared to mothers who had schedules cancelled/postpone (AOR: 4.9 (95% CI: 2.62, 9.32)).’ to ‘In comparison to children who had a vaccination postponed or cancelled, children who had not had a previous vaccination postponed or cancelled had an increased odds of receiving MCV2 (95% CI: 2.62, 9.32)).’

**Response:** Thank you, we correct it, see line 251 to 254

8. Change ‘In comparison to children whose mothers had low awareness regarding measles vaccination, children whose mothers had high awareness had an increased odds of receiving MCV2 (AOR:3.3 (95% CI: 2.12, 257 5.17)).’

**Response:** Thank you we correct it as you stated above, see line 254 to 256

9. Change ‘Similarly, the odds of vaccinating their children for MCV2 were 5 times (AOR: 2.62, 9.32) higher among children of mothers who had a good perception of measles vaccination compared to mothers with a poor perception (Table 5).’ to ‘In comparison to the children of mothers with a poor perception of measles vaccination, the children of mothers with a good perception of measles vaccination had an increased odds of receiving MCV2 (AOR: 2.62, 9.32)’.

**Response:** Thank you more, we also correct it as you stated above, see line 256 to 258

**Discussion**

1. Change ‘However, it is lower than the rates recorded Gondar in Ethiopia’ to ‘However, it is lower than the rates recorded in Gondar, Ethiopia’
1. **Response:** Thank for your critical insight, we correct as ‘However, it is lower than the rates recorded in Gondar, Ethiopia.’ See line 269
2. Start a new paragraph at ‘The disparities noted, especially in China and Japan,’ and change text to ‘The disparities noted, especially in comparison to China and Japan,’

**Response:** Thanks, corrected, as your recommendation

3. Start a new paragraph at ‘In contrast, the coverage reported in this study’

**Response:** Thank you, we start a new paragraph at ‘In contrast, the coverage reported in this study…’ See line 276

**Limitations of study:**

1. Can the number and % of children who did not have vaccination cards be described. The authors note that relying on parental recall may introduce bias which is true. To estimate the size of what effect of this bias we need to know for how many children the vaccination card was not available.

**Response:** Thank you, we describe it in % now under the methods section, see line 145

**Figures**

1. Figure 2 is unnecessary. This data can be presented in one sentence.

**Response:** Thank you for your comment. We included Figure 2 because presenting the overall coverage of MCV2 was one of our study objectives, and we felt that the figure would provide a visual emphasis. While the coverage is also summarized in the text “The overall coverage of MCV2 among children aged 19–35 months in Debre Berhan city was 57.3% (95% CI: 53.5–61.2)”, we believe the figure enhances reader understanding. Nevertheless, we are willing to remove it if the you still consider it unnecessary.

2. Figure 3. Correct the spelling errors in the title

**Response:** Thank you for your comment, I correct it accordingly.
